# Murine cutaneous responses to the rocky mountain spotted fever vector, *Dermacentor andersoni*, feeding

**DOI:** 10.3389/fmicb.2014.00198

**Published:** 2014-05-07

**Authors:** Dar M. Heinze, J. Russ Carmical, Judith F. Aronson, Franscisco Alarcon-Chaidez, Stephen Wikel, Saravanan Thangamani

**Affiliations:** ^1^Department of Pathology, University of Texas Medical BranchGalveston, TX, USA; ^2^Department of Biochemistry and Molecular Biology, University of Texas Medical BranchGalveston, TX, USA; ^3^Department of Medical Sciences, Quinnipiac UniversityHamden, CT, USA; ^4^Institute for Human Infections and Immunity, University of Texas Medical BranchGalveston, TX, USA; ^5^Galveston National Laboratory, University of Texas Medical BranchGalveston, TX, USA

**Keywords:** tick, tick feeding, tick saliva, Immunomodulation, *Dermacentor andersonii*

## Abstract

Tick salivary glands produce complex cocktails of bioactive molecules that facilitate blood feeding and pathogen transmission by modulating host hemostasis, pain/itch responses, wound healing, and both innate and adaptive immunity. In this study, cutaneous responses at *Dermacentor andersoni* bite-sites were analyzed using Affymetrix mouse genome arrays and histopathology at 12, 48, 96 and 120 h post- infestation (hpi) during primary infestations and 120 hpi during secondary infestations. The microarray data suggests: (1) chemotaxis of neutrophils, monocytes, and other cell types; (2) production and scavenging of reactive oxygen species; and, (3) keratin- based wound healing responses. Histological analysis supported the microarray findings. At 12 hpi, a mild inflammatory infiltrate was present in the dermis, especially concentrated at the junction between dermal connective tissue and underlying adipose tissue. A small lesion was located immediately under the hypostome and likely represents the feeding “pool.” Surprisingly, at 48 hpi, the number of inflammatory cells had not increased from 12 hpi, perhaps mirroring the reduction in gene expression seen at this time point. The feeding lesion is very well defined, and extravasated erythrocytes are readily evident around the hypostome. By 96 hpi, the inflammatory infiltrate has increased dramatically and the feeding lesion appears to have moved deeper into the dermis. At 120 hpi, most of the changes at 96 hpi are intensified. The infiltrate is very dense, the epidermis is markedly thickened, the feeding lesion is poorly defined and the dermal tissue near the hypostome appears to be loosing its normal architecture. In conclusion, during *D. andersoni* feeding infiltration of inflammatory cells increases across time concurrent with significant changes in the epidermal and dermal compartments near the feeding tick. The importance of changes in the epidermal layer in the host response to ticks is not known, however, it is possible the host attempts to “slough off” the tick by greatly increasing epithelial cell replication.

## Introduction

Hard (Ixodid) ticks are important vectors of disease world- wide, making them a significant threat to public health. The process of tick feeding is an extended interplay between the tick, tick salivary molecules, and the host response. Tick saliva is a complex mixture of molecules that has been shown to inhibit a broad range of host responses and facilitate pathogen transmission, while host responses can, in some cases, inhibit the feeding process and block pathogen transmission (Titus et al., 2006; Kazimírová and Štibrániová, 2013; Wikel, 2013) Hard ticks have been divided into two main phylogenetic lineages, the prostriates (containing the genus Ixodes) and the metastriates (containing all other hard tick genera). The divergence between these lineages is estimated to have occurred 124 million years ago (range from 101 to 166) (Mans et al., 2012). While salivary proteins from prostriate and metastriate lineages differ in amino acid composition, they accomplish similar goals in terms of inhibiting host responses to allow successful blood feeding. However, some differences are likely based on differential vectorial capacity. In this study, cutaneous host responses to *D. andersoni* were measured in an effort to understand host factors effecting pathogen transmission. Affymetrix GeneChip Mouse Genome 430A 2.0 arrays were used to measure host gene expression in the skin of *D. andersoni* infested mice at 12, 48, 96, and 120 h post- infestation (hpi) during primary infestations and 120 hpi during secondary infestations. In addition, histopathological analysis of bite-site lesions from primary infestation time points and quantitative real-time PCR analysis of lymph nodes from secondary infestation time points were analyzed. These studies allow us to describe the cutaneous host response during primary and secondary infestations, measure changes in gene expression patterns across time, view potential patterns related to tick immuno-suppression, correlate the histopathology analysis to the gene expression data, and measure systemic responses in the draining lymph node. These analyses are important for understanding the context of pathogen transmission and tick rejection at the tick-host inGAterface.

## Methods

### Ticks

Pathogen-free *D. andersoni* colonies were maintained in our laboratory as described (Bouchard and Wikel, 2005; Heinze et al., 2012a,b). All life cycle stages were kept in sterile glass vials with mesh tops in desiccators at 22°C containing saturated solutions of KNO_3_ to obtain desired relative humidity with a 16:8 h photoperiod. For routine colony maintenance adult and/or nymphal ticks were fed on New Zealand white rabbits and nymphs and/or larvae were fed on mice.

### Animals

BALB/c mice used in this study were obtained from The Jackson Laboratory (Bar Harbor, ME). Mice were cared for in accordance with a protocol approved by the Institutional Animal Care and Use Committee (IACUC) of the University of Texas Medical Branch.

### Time course infestations

To perform time course infestations, 6–8 week old female BALB/c mice were placed into individual restrainers or anesthetized with a 150 μl intraperitoneal injection containing 10 mg/mL ketamine (Fort Dodge Animal Health, Fort Dodge, IA) and 1 mg/mL xylazine (Phoenix Pharmaceutical, St. Joseph, MO) in sterile PBS (Gibco, Life Technologies, Carlsbad, CA) and infested with pathogen-free nymphal ticks. Ticks were allowed to attach for approximately 1 h and unattached ticks were discarded. Mice were then removed from restraints and housed individually. Secondary infestations involved two rounds of infestation. Mice were infested with nymphal ticks that were allowed to complete their feeding cycle (4–5 days). 14 days after the last primary infestation tick completed feeding, mice were re-infested with nymphal ticks using the same protocol described above. Bite sites were analyzed at 12, 48, 96, and 120 hpi during primary infestations, and at 120 hpi during secondary infestations. Three mice were measured at each time point in all micro-array or PCR-array experiments; controls consisted of 3 similarly housed but tick-free mice. Skin biopsies (4 mm) at the tick feeding loci (around ear) were harvested and stored in RNAlater (Ambion) at −20°C for RNA extraction or fixed in 10% neutral buffered formalin for 48 h and then stored in 70% ethanol at 4°C for histopathology. For histopathology, skin biopsies were harvested with ticks attached.

### RNA isolation

Ticks were removed from all skin biopsies before RNA extraction. Tissue samples were homogenized individually in 1 mL Trizol (Life Technologies, Carlsbad, CA) using an Ultra-Turrax T8 (Ika, Wilmington, NC) tissue disperser. The Trizol protocol was followed until phase separation. The aqueous phase was retained and one volume 70% ethanol (Acros Organics) was added and the samples applied to RNeasy micro kit (Qiagen) columns. The RNeasy protocol was then followed, including the in-column DNase digestion step. One extra wash step with buffer RW1 and two extra wash steps with buffer RPE facilitated the isolation of high quality RNA. All samples were eluted in nuclease-free water. After extraction, RNA was quantitated spectrophotometrically using a NanoDrop ND-1000 (NanoDrop Technologies, DE). All samples were required to read greater than 1.8 on both A_260_/A_280_ and A_260_/A_230_ ratios. For subsequent microarray analysis, quality of the purified RNA was assessed by visualization of 18 and 28 S RNA bands using an Agilent BioAnalyzer 2100 (Agilent Technologies, CA). Resulting electropherograms were used in the calculation of the 28.18 S ratio and the RNA Integrity Number, which was greater than 6.8 in all samples (Schroeder et al., 2006). For subsequent real-time PCR analysis, RNA integrity was determined by denaturing (formaldehyde) agarose gel electrophoresis followed by staining with Sybr Gold stain (Invitrogen). Visualization of clear ribosomal bands indicated minimal degradation. Eluted RNA samples were aliquoted and stored at −80°C until used.

### Host gene expression profiling using affymetrix genechips

**Figure 1:**
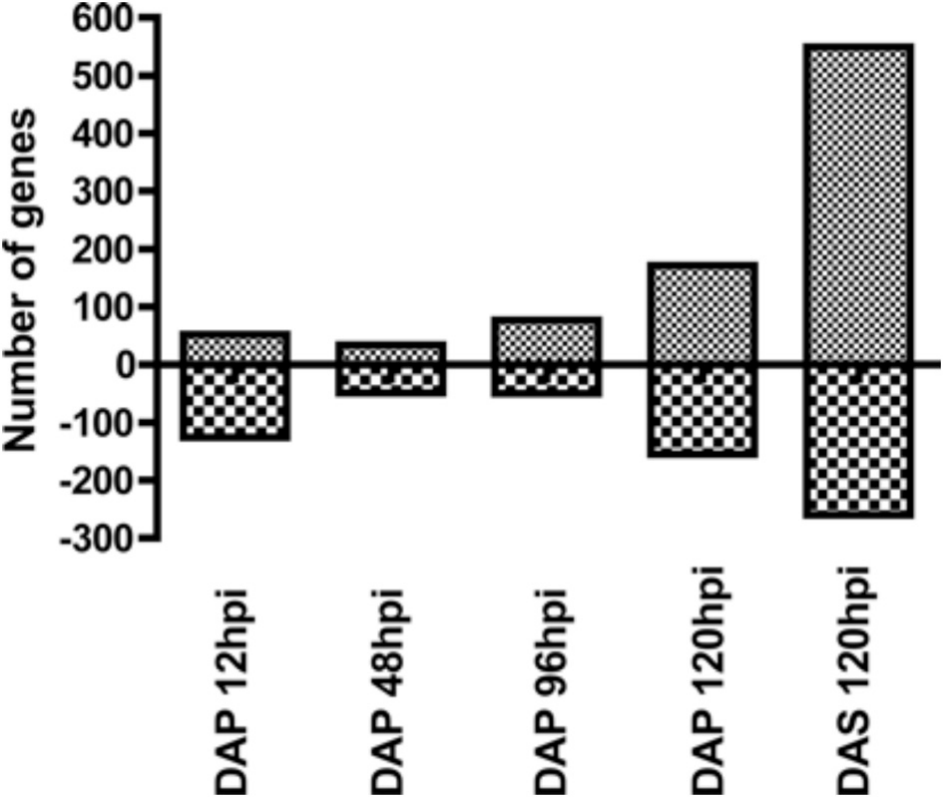
**Number of up and downregulated genes at each time point in the microarray data**. Mice were infested with *D. andersoni* nymphs and 4 mm skin biopsies collected at 12, 48, 96, and 120 hpi during primary infestations and 120 hpi during secondary infestations. Gene expression profiling was measured using Affymetrix mouse genome 430A 2.0 arrays. Significance was assessed using iReports data analysis methods, and results were filtered for genes with fold changes greater than ±1.5 and *p*-values less than 0.05. DAP, *D. andersoni* primary; DAS, *D. andersoni* secondary; hpi, hours post infestation.

Total RNA (500 ng) was converted to cRNA for microarray analysis using the Ambion MessageAmp™ Premier RNA Amplification Kit (Life Technologies Corporation, CA) according to manufacturer's instructions. Total fragmented cRNA (10 υ g) was hybridized to the Affymetrix GeneChip Mouse Genome 430A 2.0 array according to the manufacturer's (Affymetrix, CA) conditions. The chips were washed and stained in a GeneChip Fluidics Station 450 and fluorescence detected with an Affymetrix-7G Gene Array scanner using the Affymetrix GeneChip Command Console software (AGCC1.1). The resulting CEL files were uploaded to iReport™ (Ingenuity Systems), and the default data analysis was utilized. The resulting values were then filtered for *p*-values ≤ 0.05 and a fold change ≤ −1.5 or ≥ +1.5 and these data are reported as significant findings (Figure 1).

### Gene ontology(GO) analysis

**Figure 2:**
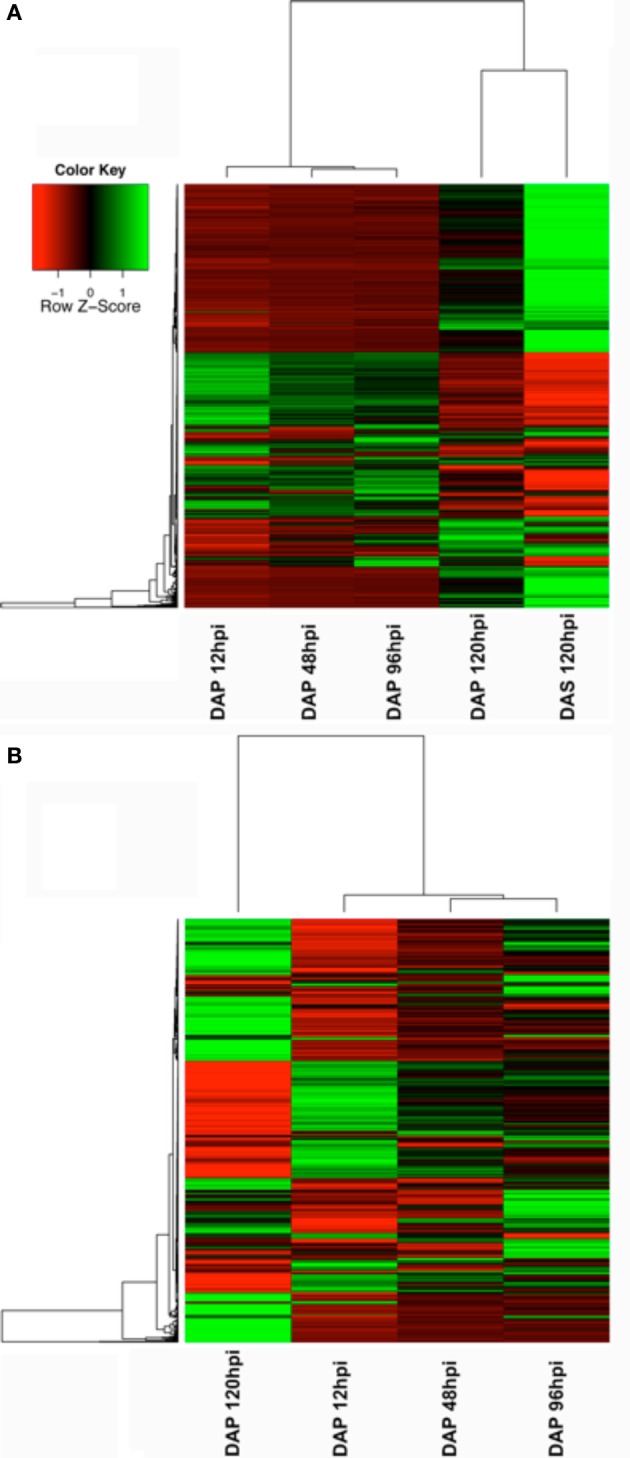
**Heatmaps showing changes in gene expression across time**. **(A)** Changes in genes significant at any time point in the microarray study. **(B)** Changes in genes significant during the primary infestation only.

**Figure 3:**
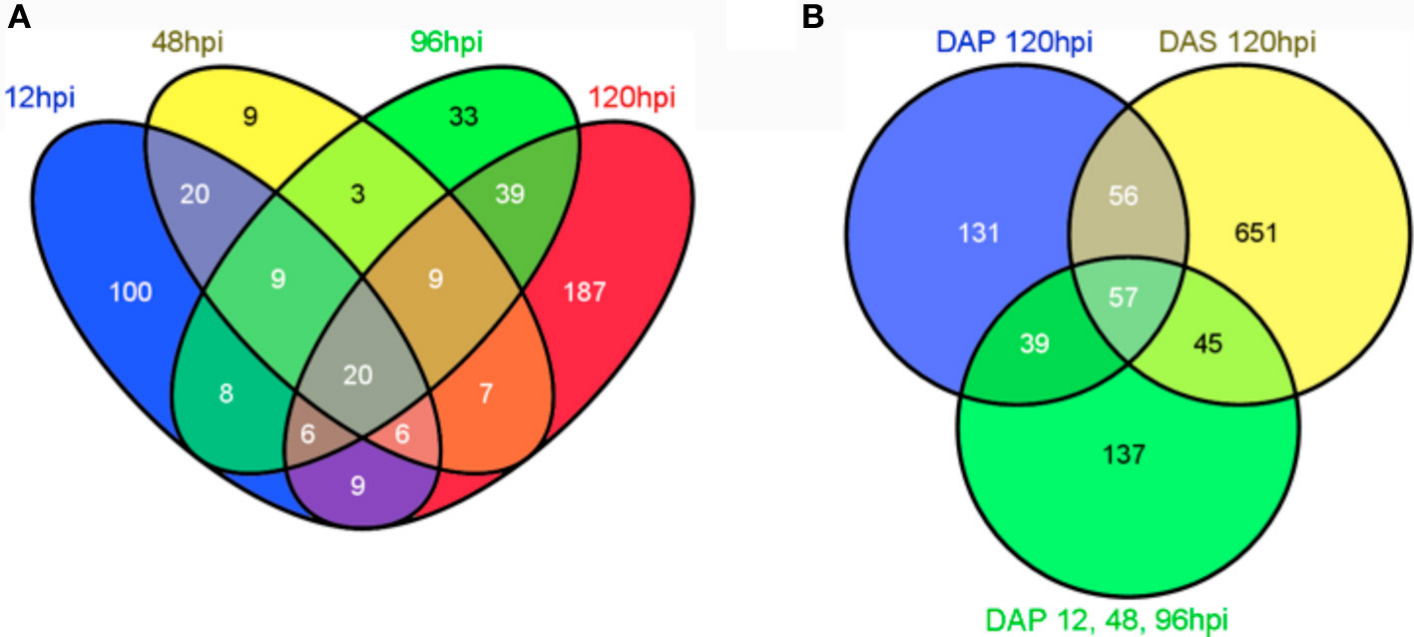
**Venn diagram showing the overlap of significantly modulated genes between time points in the microarray study. (A)** Venn diagram for primary infestation time points. **(B)** Venn diagram for early time points (DAP12, 48, and 96 hpi), later time points (DAP120 hpi), and secondary infestation (DAS120 hpi). The Venn diagram in B was constructed based on variations in gene expression profiles suggested in the heatmaps. All Venn diagrams were created using (Oliveros, 2007).

Broad patterns of significantly modulated genes were visualized using heatmaps plotted in the R package gplots (Warnes et al., 2013). Two gene lists were used to generate these maps; the first included all genes modulated during the primary infestation and the second included all genes modulated at any time point in the study (Figure 2). These heat maps suggested modulated genes grouped into early primary infestation (12–96 hpi), late primary infestation (120 hpi), and secondary infestation patterns (120 hpi). The significantly modulated genes in these groups were compiled and used to generate a Venn diagram (Figure 3) through an online interactive tool (Oliveros, 2007). The lists of unique and shared genes in each group identified in the Venn diagram were analyzed using the Database for Annotation, Visualization, and Integrated Discovery (DAVID) (Huang da et al., 2009a,b) website using the Affymetrix GeneChip Mouse Genome 430A 2.0 array as a background. The functional annotation clustering tool was used to group similar gene ontology terms into clusters with a mathematically-generated ranking score. These clusters can then be named based on the gene ontology terms in each cluster, giving a concise over-view of the results (Table 1).

**Table 1:** **Top ten clusters from DAVID analysis based on lists from Figure 3B Venn diagram**.

**Core**		**DAP12-48-96 only**	
1	Chemotaxis, immune response, response to wounding	1	Response to DNA damage and cellular stress, DNA repair
2	Chemotaxis, cell migration	2	Intracellular non-membrane-bounded organelle
3	Cation homeostasis	3	Nucleotide and ATP binding
4	Intermediate filament, cytoskeleton organization and structure	4	Regulation of transcription, DNA binding
5	Keratinization, keratinocyte and epidermal cell differentiation	5	Circadian rhythm
6	Response to metal ion	6	Chromosome, chromatin binding and organization
7	Carbohydrate binding	7	Nuclear body, nucleoplasm
8	Regulation of blood pressure	8	mRNA metabolic process
9		9	Regulation of transcription
10		10	Cardiac muscle contraction
**DAP120-DAP12-48-96**		**DAP120 only**	
1	No clusters with significant terms	1	Regulation of cell and cell-substrate adhesion
2		2	Blood vessel development
3		3	Embryonic development
4		4	Nuclear or intracellular organelle lumen
5		5	RNA processing
6		6	Cell growth
7		7	MAP kinase activity
8		8	Positive regulation of transcription and RNA metabolic process
9		9	Meisis (cell cycle)
**DAP120-DAS120**		**DAS120**	
1	Extracellular region	1	Inflammatory and defense response
2	Proteinaceous extracellular matrix	2	Leukocyte and lymphocyte activation, T cell differentiation
3	Cell surface	3	Positive regulation of immune response, immune response-regulating cell surface receptor signaling pathway
4	Response to wounding	4	Lysosome
5	Cellular homeostasis	5	T cell activation, leukocyte and lymphocyte proliferation
6	Fatty acid biosynthetic process	6	Chemotaxis
7	Regulation of phosphorylation, protein kinase activity	7	Regulation of cytokine production
8		8	Regulation of immune cells
9		9	Regulation of adaptive immune response
10		10	Endocytosis
**DAS120-DAP12-48-96**			
1	Keratinocyte, epidermal cell differentiation		
2	Rhythmic process		

The core analysis represents significantly modulated genes shared between early primary (DAP12, 48, 96), late primary (DAP120), and secondary infestations (DAS120). DAP, D. andersoni primary; DAS, D. andersoni secondary.

### Validation of array data

The GeneChip results were validated by an additional experiment. A separate set of mice were infested with ticks and tissues harvested as described above. For the D. andersoni microarray experiment, gene targets (Table 2) were validated at 12, 48, 96, and 120 hpi during primary infestations and 120 hpi during secondary infestations using 3 mice/time point. Primers were purchased from Integrated DNA Technologies; these primer sequences are provided in Table 3. Primers were mixed with RT^2^ SYBR green qPCR master mix (Qiagen) and aliquoted into iCycler iQ PCR plates (Bio-Rad) using an epMotion 5075 automated pipetting system (Eppendorf). Plates were sealed and stored at −20°C until use. For each real-time PCR run, the RT^2^ First Strand Kit (Qiagen) was used to convert 1 υ g total RNA into cDNA, which was then loaded onto PCR plates using the epMotion 5075 automated pipetting system (Eppendorf). In some cases, the epMotion system was not functioning properly and the plates were loaded with primer and/or cDNA using an 8-channel pipette. These plates were run on an iCycler iQ5 real-time PCR instrument (Bio-Rad) with the following cycling protocol: 10 min at 95°C; 15 s at 95°C, 1 min 60°C for 40 cycles, and an 80-cycle (+0.5°C/cycle) 55–95°C melt curve. Every run included hypoxanthine guanine phosphoribosyl transferase (Hprt) and heat shock protein 90 alpha (Hsp90ab1) as endogenous control genes, and “no template” and “no first strand” controls. HTqPCR, an R-based program for real-time PCR data analysis (Dvinge and Bertone, 2009), was used to analyze data using the delta-delta Ct method for gene expression normalization and measurement, and the linear models in microarray analysis (LIMMA) package for statistical comparisons between infested and tick-free mice.

**Table 2:** **Gene list for quantitative real-time PCR validation of the *D. andersoni* microarray study**.

Arg1	Cxcl5	il4	S100a9
Ccl12		Il6	sele
Ccl6	Hprt	Irak4	Serpine1
Ccl7	Hsp90ab1	krt16	Socs3
Ccr1	ifng	krt6b	Stat3
Chi3l1	il10	Map3k6	Wnk1
Clec10a	il12a	Mt1	No RT
Clec4e	Il1b	ptges	No temp

**Table 3:** **List of gene targets and primer sequences used in the *D. andersoni* quantitative real-time PCR validation of the microarray data and also in quantitative real-time PCR analysis of lymph nodes at 120 hpi during secondary infestations**.

**Gene name**	**Primer 1 (5′–3′)**	**Primer 2 (5′–3′)**
RORgt	Ccgctgagagggcttcac	Tgcaggagtaggccacattac
Gapdh	gtggagtcatactggaacatgtag	Aatggtgaaggtcggtgtg
Tbx21	caagaccacatccacaaacatc	Ttcaaccagcaccagacag
Arg1	Agtgttgatgtcagtgtgagc	Gaatggaagagtcagtgtggt
Stat4	Caactcctctgtcaccatgt	Cgtcaaagctatgtccagtga
Chi3l1	Ccatcaaagccataagaacgc	ccagaaacaccaacctgaaga
Hdc	Gaccgaatcacaaaccacag	tctacctccgacatgccaa
Irak4	Cagcagtagttgaggttcacg	Acacccaaatctgacatctaca
Stat1	gacttcagacacagaaatcaactc	Ttgacaaagaccacgcctt
Stat6	agttcttcctgcttccgatg	Gccaccatcagacaaatacttc
Tgfb1	cgtggagtttgttatctttgctg	Gacgtcactggagttgtacg
Stat3	Agctcctcagtcacgatca	gttcaagcacctgacccttag
Ptges	Caggaatgagtacacgaagcc	Gtattacaggagtgacccagatg
Il12a	ctctcgttcttgtgtagttcca	acagatgacatggtgaagacg
Foxp3	Ctgtcttccaagtctcgtctg	Ctggtctctgcaggtttagtg
Lcn2	Cctgtgcatatttcccagagt	Ctacaatgtcacctccatcctg
Il1b	Cgagatttgaagctggatgc	Tgacagtgatgagaatgacctg
Il17a	gagcttcccagatcacagag	Agactacctcaaccgttcca
Il10	Atggccttgtagacaccttg	Gtcatcgatttctcccctgtg
Sele	Cctgattgttttgaacctagacg	Cgtcctcattgctctacttgt
Stat5a	Cgcttgattcttttcagtgaca	Tgagaacacccgcaatgag
Ccl6	Gaagtgtcttgaaagccttgatg	Agaaactccaagactgccat
Hprt	Aacaaagtctggcctgtatcc	Ttccctggttaagcagtacag
Serpine1	ggctgagatgacaaaggct	Tcacaagtctttccgaccaag
Il6	Gcaagtgcatcatcgttgttc	Agtcggaggcttaattacacat
Gata3	gtccccattagcgttcctc	Ccttatcaagcccaagcgaa
Ifng	Agataatctggctctgcagga	Gtcattgaaagcctagaaagtctg
Hsp90ab1	Cctgaaaggcaaaggtctcc	Ccaccctgctctgtactact
Clec10a	gaccaaggagagtgctagaag	tgactgagttcctgcctct
Wnk1	Aaaggcatggttcaaaaggtc	Gcagatctaccgtcgagtga
Ccr1	Aggaactggtcaggaataatagc	Caaaggcccagaaacaaagtc
Il4	Tgatgctctttaggctttccag	Cagagactctttcgggcttt
Clec4e	Cttatggtggcacagtcctc	Agtggcaatgggtggatg
S100a9	ccatcagcatcatacactcctc	Tggaagcacagttggcaa
Map3k6	Gatttccggggccatatactg	Cacagagacatcaagggagac
Krt6b	Ctgcttttgtacgcttgttga	Gacagcatcattggagagagg
Ccl7	Tttgtttcttgacatagcagcat	Tctcactctctttctccacca
Cxcl5	Gatccagacagacctccttct	ttgatcgctaatttggaggtga
Mt1	gctcttcttgcaggaggtg	Tcacttactccgtagctccag
Krt16	cagctcattctcgtacttggtc	Tcaaagactacagcccctact
Actb	Cgatggaggggaatacagc	Tctttgcagctccttcgtt
Socs3	ggaaacttgctgtgggtga	Gagatttcgcttcgggacta
Ccl12	Ggaggcatagaagtgtggaaa	agagacactggttcctgact

### Quantitative real-time PCR analysis of mouse lymph nodes after *D. andersoni* feeding

Draining cervical/auricular lymph nodes were harvested from mice used for validation of the *D. andersoni* microarray data set during secondary infestations at 120 hpi. Quantitative real-time PCR analysis was undertaken using the gene targets in Table 4. These results were compared against lymph nodes from tick-free mice. RNA was isolated and PCR run as described above.

**Table 4:** **Gene list for quantitative real-time PCR analysis of mouse lymph nodes after secondary infestation with *D. andersoni* for 120 h**.

Actb	Il12a	Stat3
Foxp3	Il17a	Stat4
Gapdh	Il1b	Stat5a
Gata3	Il4	Stat6
Hprt	Il6	Tbx21
Hsp90ab1	RORgt	Tgfb1
Ifng	Socs3	No RT
Il10	Stat1	No temp

## Results and discussion

The number of up and downregulated genes meeting our filtering criteria at each time point is shown in Figure 1. Although there was a small decrease in the number of upregulated genes between 12 and 48 hpi, the general trend was an increasing number of upregulated genes across time. The downregulated genes show a different profile, with more genes modulated at early and late time points than those in the middle. A list of all genes modulated at any time point (Supplement data 1) in the study was used generate a heatmap (Figure 2A). This heatmap suggested a similar gene expression profile for the first three time points (12, 48, and 96 hpi during primary infestations) that then shifted to a second pattern that was similar between 120 hpi in primary and secondary infestations. This suggested there was a shift in gene expression profiles between early and late time points, as well as between primary and secondary infestations.

To provide a higher-resolution analysis of the primary infestation time points, a second heatmap was constructed using only the significantly modulated genes in the primary infestation (Figure 2B). While this heatmap shows some differences between 12, 48, and 96 hpi, the overall similarity seen in the first heatmap was preserved. In addition, the dramatic change in expression profile between the first three time points and the 120 hpi primary infestation time point was maintained.

A Venn diagram for the primary infestation time points (Figure 3A) shows more uniquely modulated genes at 12 hpi and 120 hpi than at other time points, again suggesting differences between early and late responses. To explore the patterns observed in the heatmaps, all significantly modulated genes from 12, 48, and 96 hpi primary infestation were combined into a single data set. This list, the list of modulated genes at 120 hpi primary infestation, and the list of modulated genes at 120 hpi secondary infestation were used to create a Venn diagram (Figure 3B). This revealed 137 genes unique to early primary infestations, 131 genes unique to late primary infestations, 651 genes unique to late secondary infestations, and 57 genes shared across all time points. These lists, along with the lists of genes shared between each group were submitted to the Database for Annotation, Visualization, and Integrated Discovery (DAVID) bioinformatics database using the Affymetrix Mouse Genome 430A 2.0 array as a background list. The functional annotation-clustering tool was used to group similar ontology terms together, and the top ten clusters containing significant ontology terms (*p* < 0.05) were named based on the terms included (Table 1).

### The core response (genes shared between all time points)

Analyzing the shared response between all time points allows the description of a “core” response to tick feeding. Genes in the core response were significant at early, primary, late primary, and late secondary. Significant gene ontology clusters were chemotaxis and inflammation, cation homeostasis, intermediate filaments and keratinization, carbohydrate binding, and regulation of oxidatives stress.

Genes in the chemotaxis and inflammation clusters were chemokines, cytokines, and anti-microbial molecules. Chemokines Ccr1, Ccl2, Ccl6, Ccl7, Ccl12, Cxcl1, Cxcl2, and Pf4 (platelet factor 4 or Cxcl4) were upregulated and are consistent with monocyte and neutrophil migration into the bite site. Additional chemoattractants for neutrophils such as the S100 molecules (calgranulins) and Saa3 (serum amyloid A3) were also upregulated. Antimicrobial proteins such as Lcn2 (lipocalin 2) and Ptx3 (pentraxin related gene) that function in iron sequestration and opsonization respectively were upregulated. These results combined with the upregulation of IL-1b suggest a potent innate-like immune response dominated by macrophages and neutrophils is a hallmark of the anti-tick response.

Gene ontology terms in clusters 3 and 6 were related to cations or metal ions. Genes in these clusters related to ion regulation were Mt1 and Mt2 (metallothionein); these molecules are involved in regulating copper and zinc, but also are involved in reactive oxygen species scavenging, cell transcription, and immune responses. Studies with metallothionein knockout mice suggest Mt1 and 2 play protective roles in inflammatory settings (Inoue et al., 2009; Thirumoorthy et al., 2011). Xanthine dehydrogenase, an enzyme involved in the oxidative metabolism of purines, was also upregulated. Xanthine dehydrogenase may be converted to xanthine oxidase by sulfhydryl oxidation, and xanthine oxidase can be an important source of reactive oxygen species (Sakuma et al., 2012). Thus the generation and scavenging of reactive oxygen species appears to be the primary function of the genes in these clusters.

Clusters 4 and 5 were dominated by gene ontology terms related to intermediate filaments and keratinization. Indeed, keratin intermediate filaments Krt6a, 6b, and 16 were upregulated. Krt16 has been shown to pair with either Krt6a or 6b to form essential intermediate filaments in epithelial wound repair. Specifically, these intermediate filaments are required to support keratinocyte migration into the wound site (Paladini et al., 1996; Wong and Coulombe, 2003). In addition to these molecules, Sprr1b (small proline rich 1b) was upregulated. The encoded protein is a significant component of the cornified cell envelope that maintains a permeability barrier across epithelial tissues (Patel et al., 2003). Downregulated genes included a minor collagen (type 11 alpha 1), the cytoskeletal component spectrin, and a little-studied molecule Limch1 (LIM and calponin homology domains 1). These results suggest a keratin intermediate filament-based wound healing response is activated at the tick feeding lesion, however it is surprising that other cytoskeletal elements are downregulated, a pattern that is even more striking in the early primary infestation as discussed below. Thus keratin intermediate filaments but not other cytoskeletal molecules are activated in response to tick bites.

Gene ontology cluster 7 was related to carbohydrate binding. The primary molecules in this group were c-type lectin receptors Clec4d and Clec4n, although other molecules such as Ccl7, Pf4, and Ptx3 were also present. C-type lectins are expressed on dendritic cells and macrophages, and play important roles in orchestrating the inflammatory response. Their importance host responses to tick feeding is strongly suggested by studies showing the *I. scapularis* salivary protein Salp15 can bind and activate DC-sign (CD209) (Hovius et al., 2008). Clec4n (dectin-2) is thought the associate with FcRγ and signal through a Syk-Card9 pathway to activate NFkB (Geijtenbeek and Gringhuis, 2009). Ligands include mannose, fungal antigens, and dust mites (Patel et al., 2003), although others likely exist. The precise role of c-type lectins in tick feeding is unknown, but these results suggest they may act as important pattern recognition receptors and may modulate downstream responses.

The final cluster contained terms related to regulation of blood pressure. This primary gene in this group, Wnk1 (lysine deficient protein kinase 1), is downregulated and it influences blood pressure by regulating salt absorption in the kidney. Wnk1 also plays a similar role in sweat glands (Kahle et al., 2008). Upregulated genes in this group include Hmox1 (heme oxygenase 1), and Gch1 (GTP cyclohydrolase 1). Hmox1 can play a role in protecting tissues from oxidative damage, while Gch1 can produce either NO or reactive oxygen species depending cofactor availability. Thus the gene ontology terminology may be misleading here, as the genes appear more related to regulating oxidative status than blood pressure.

### Early primary infestation

Gene ontology analysis of the early primary infestation revealed a number of clusters related to nucleotide processing such as DNA repair, nucleotide binding, transcription, and mRNA metabolism. A review of the genes represented by these clusters suggested they could be broadly grouped into DNA repair molecules, DNA helicases of the SWI/SNF family, transcription factors, components of the spliceosome, and mRNA metabolism. DNA helicases of the SWI/SNF family are not active helicases, but function as DNA translocases thought to modulate chromatin structure (Durr et al., 2006). Thus most of these molecules can be related to transcription, from chromatin remodeling, transcription factor binding, RNA splicing, and mRNA metabolism. Interestingly, nearly all of these genes were downregulated, suggesting that transcription is decreased early in the primary infestation. Of the few upregulated transcriptional regulators, Nfkbia and Tsc22d3 were of particular interest because they have been shown to inhibit NFkB and AP-1 pro-inflammatory pathways (Beaulieu and Morand, 2011). DNA repair molecules were also downregulated. In contrast, molecules in the circadian rhythm cluster, such as Per1 were upregulated. Overexpression of Per1 in tumor cells was shown to increase DNA-damage induced apoptosis and significantly decreased cell growth in untreated cells (Gery et al., 2006). This suggests some connection between DNA repair, circadian rhythm molecules, and growth arrest in the skin at the bite site may exist. Terms in the final cluster were related to cardiac muscle contraction, although review of these genes and genes in the highly significant (though un-clustered) GO term actin binding (GO:0003779) revealed molecules related to muscle contraction, actin binding, and cytoskeletal adaptor molecules that may relate signaling events to changes in the cytoskeleton. Cytoskeletal elements have been shown to play vital roles in epithelial wound healing processes by aiding cell migration and wound contracture (Abreu-Blanco et al., 2012). Thus the early host responses were characterized by a reduction in transcription, DNA repair, inhibition of inflammation, potential inhibition of the cell cycle, and downregulation of cytoskeletal elements. These effects suggest that tick feeding may inhibit early aspects of the wound healing response.

When the genes shared between early and late primary infestation were submitted to DAVID, no significant clusters were observed.

### Late primary infestation

The first three clusters (regulation of adhesion, blood vessel development, embryonic development) and clusters 6 and 7 (cell growth, MAPK activity) share similar genes and are treated together here. In the process of normal epidermal wound healing, repair must take place in the epidermis, dermis, and vasculature to restore normal function. Particularly for the re-epithelialization of the wound, keratinocytes must loose some of their adhesiveness and migrate into the wound area. This process shares similar characteristics to the epithelial-mesenchymal transition (EMT) (Nakamura and Tokura, 2011) during embryonic development. In support of a similar process at the tick bite site, genes known to be involved in EMT such as Tdgf1 (teratocarcinoma-derived growth factor 1 or Cripto-1), Cyr61 (cysteine rich protein 61 or CCN1), and Smad5 (SMAD family member 5, a TGF-β signaling intermediate) were upregulated. While not specific to EMT, the MAPK signaling pathway is known to be important (Leopold et al., 2012), increasing the relevance of cluster 7. Additional upregulated genes that may be involved were Tnfrsf12s (tumor necrosis factor receptor superfamily, member 12a), Junb (Jun-B oncogene), and Epgn (epithelial mitogen). While a specific role for the encoded proteins in EMT has not been delineated, these molecules may be related to TNF-α, AP-1, and growth factor responses that are known to be involved (Leopold et al., 2012).

Downregulated molecules included extracellular matrix components Col1a1 (collagen type I alpha 1) and Lamb2 (laminin beta 2), calcium ion channel or signaling molecules Tesc (tescalcin) and Pkd2 (polycyctic kidney disease 2), integrin ligand Edil3 (EGF-like repeats and discoiding I-like domains 3), and growth factor related molecules Pdgfrb (platelet derived growth factor receptor beta) and Tgfb3 (transforming growth factor beta 3). In the final analysis, a convincing case for EMT in wound healing responses during late primary infestations cannot be made. However, changes in adhesion, cell growth, MAPK activity, and the appearance of genes normally associated with embryonic development such as Tdgf1 are suggestive of a related process.

The remaining clusters were nuclear or organelle lumen, RNA processing, positive regulation of transcription, and meiosis or cell cycle related. The majority of the genes in these clusters code for RNA binding proteins, RNA processing proteins, and transcriptional activators/transcription factors. An interesting theme among the RNA-related molecules was an interaction with rRNA, implying some interaction with ribosomes. Most of the genes in these clusters were upregulated, contrasting with the transcription-related genes early in the primary infestation. Thus, the data suggests transcription is inhibited during early host responses, but then activated near the end of the feeding cycle.

Genes shared between 120 hpi primary and 120 hpi secondary infestation clustered into a number of broad categories such as extracellular region, cell surface, cellular homeostasis, and regulation of phosphorylation. Two clusters were more specific, however. Fatty acid biosynthesis contained three genes, Fcer1a (IgE Fc receptor), Scd3 (stearoyl-coenzyme A desaturase), and Ch25h (cholesterol 25-hydroxylase). Fcer1a and Ch25h were upregulated, while Scd3 was downregulated. Fcer1a encodes the alpha chain of the FceR, a receptor that binds IgE with high affinity. This receptor is activated by antigen-induced cross-linking, and induces many downstream responses including the release of histamine and the production of prostaglandins D2 and E2 (Ugajin et al., 2011). Supporting this role, genes encoding proteins involved in prostaglandin E synthesis are upregulated at 120 hpi primary infestation, although they are not shared with 120 hpi secondary infestation.

Ch25h encodes a protein that catalyzes the formation of 25-hydroxycholesterol (Lund et al., 1998), a molecule that can act as a chemoattractant for migrating cells but also inhibits the function of B cells (Diczfalusy et al., 2009) and can promote the survival of Listeria monocytogenes infected cells (Zou et al., 2011). Scd3 is an important enzyme in the synthesis of monounsaturated fatty acids, many of which are subsequently used as components of membrane phospholipids. Interestingly, decreasing fatty acid synthesis inhibited rift valley fever virus replication in mouse embryonic fibroblasts (Moser et al., 2012), suggesting fatty acid synthesis may aid viral replication. These genes suggest a role for fatty acid metabolites and the control of fatty acid synthesis at the tick bite site. Finally, the response to wounding cluster contained pro-inflammatory genes such as Il-6, Cxcl5, and P2ry12 (purinergic receptor P2Y) and extracellular matrix molecules Cd44 and Timp3 (tissue inhibitor of metalloproteinase 3). These genes are involved in inflammation and cell migration, although they are often associated with acute inflammation rather than appearing late in the induction of the immune response as they do here.

In summary, late in the primary infestation the innate immune response remains the major player. Keratin-based wound healing responses and the suggestion of an EMT-type transition driving wound re-epithelialization are present. In addition, transcription appears to be potentiated in contrast to earlier in the primary infestation. Genes shared with the secondary infestation suggest fatty acid metabolism and the response to wounding are important in host responses near the end of the feeding cycle.

### Secondary infestation

There were 651 genes modulated only in the secondary infestation. Gene ontology analysis of this group of genes resulted in many clusters related to the immune response. These clusters shared many genes with each other and they will be discussed in relation to what appeared to be the most distinctive feature(s) of each cluster or group of clusters.

The first cluster contained GO terms related to inflammatory and defense responses. Genes in this group supported four primary processes: complement (synthesis), coagulation (pathway synthesis), Toll-like receptor/IL-1 response, and acute phase response. Clusters 2, 5, 8, and 9 were leukocyte and lymphocyte activation, T cell activation and leukocyte proliferation, regulation of immune cells, and regulation of adaptive immune response, respectively. These clusters shared similar genes, and supported the importance of protein kinases (SRC family, Syk, and Jak), T-cell costimulation (CD40, CD86, CD3d, and Lck), cytokines, immunoreceptors (cytokine, antibody, C-type lectin, and Toll-like receptors 1 and 4), and signaling intermediates in immunoreceptor signaling. Cluster 3, positive regulation of immune response, contained many similar features. However, two genes strongly related to iron handling in the innate immune response, Slc11a1 (solute carrier family 11 member 1) and Hpx (hemopexin) were upregulated. Cluster 4 contained GO terms related to lysosomes. The largest group of upregulated molecules in this cluster were genes encoding many different cathepsins. Cathepsins are lysosomal proteinases that function primarily in protein turnover in the lysosome (Kuester et al., 2008). However, additional roles for cathepsins include (but are not limited to) antigen presentation (Yamamoto et al., 2012), keratinocyte differentiation (Yamamoto et al., 2012), itch responses (Andoh et al., 2012), and wound healing (Akira and Takeda, 2004). The chemotaxis cluster contained chemokines Ccl24, Ccl17, Ccl5, Ccl4, Ccl8, Ccl3, and Cxcl16 consistent with the migration of lymphocytes, monocytes, neutrophils, eosinophils, and basophils into the bite site. Additional molecules that supported cell migration such as integrins, chemoattractants, and intracellular molecules that regulate changes in cell shape were also upregulated. Cluster 7 contained terms related to regulation of cytokine production. These genes code for proteins that function as immunoreceptors, cytokine signaling intermediates, and cytokines. Finally, the endocytosis cluster contained genes encoding classical endocytotic proteins such as Clta (clathrin, light polypeptide) and picalm (phosphatidylinositol binding clathrin assembly protein). Other genes in this cluster were antibody Fc receptors Fcer1g, Fcgr3, and Fcgr2b and lectin recepors Clec7a, CD209a CD209d, CD209e, and Mrc1. These receptors may function in endocytosis by binding antibodies or carbohydrate moieties and triggering endocytotic machinery. This may be an important method of delivering tick-derived antigens to the endosomal/lysosomal compartment where they could be processed for antigen presentation.

These results suggest the importance of complement, coagulation, innate immune responses, and adaptive immunity in the host response to *D. andersoni* nymphs. In particular, the importance of Toll-like receptors 1 and 4 and lectin pattern recognition receptors appear to be important aspects of the innate immune response during secondary infestations. Both TLRs and C-type lectins have been shown to be important pattern recognition receptors for sensing pathogens and initiating and shaping innate and adaptive immune responses (Akira and Takeda, 2004; Geijtenbeek and Gringhuis, 2009). Both TLR2 and DC-SIGN (CD209e) have already been shown to be important in anti-tick responses by modulating cytokine responses (Hovius et al., 2008; Oliveira et al., 2010). These studies support our results and also suggest the importance of these receptors is likely broader than previously reported. Within the adaptive immune response, these results suggest the capture, processing, and presentation of antigens is an important process during secondary infestations. In particular, cathepsins may be important proteins in this process with possible contributions to wound healing and/or itch responses. The array results also support the co-stimulation and activation of lymphocytes.

### Validation

**Figure 4:**
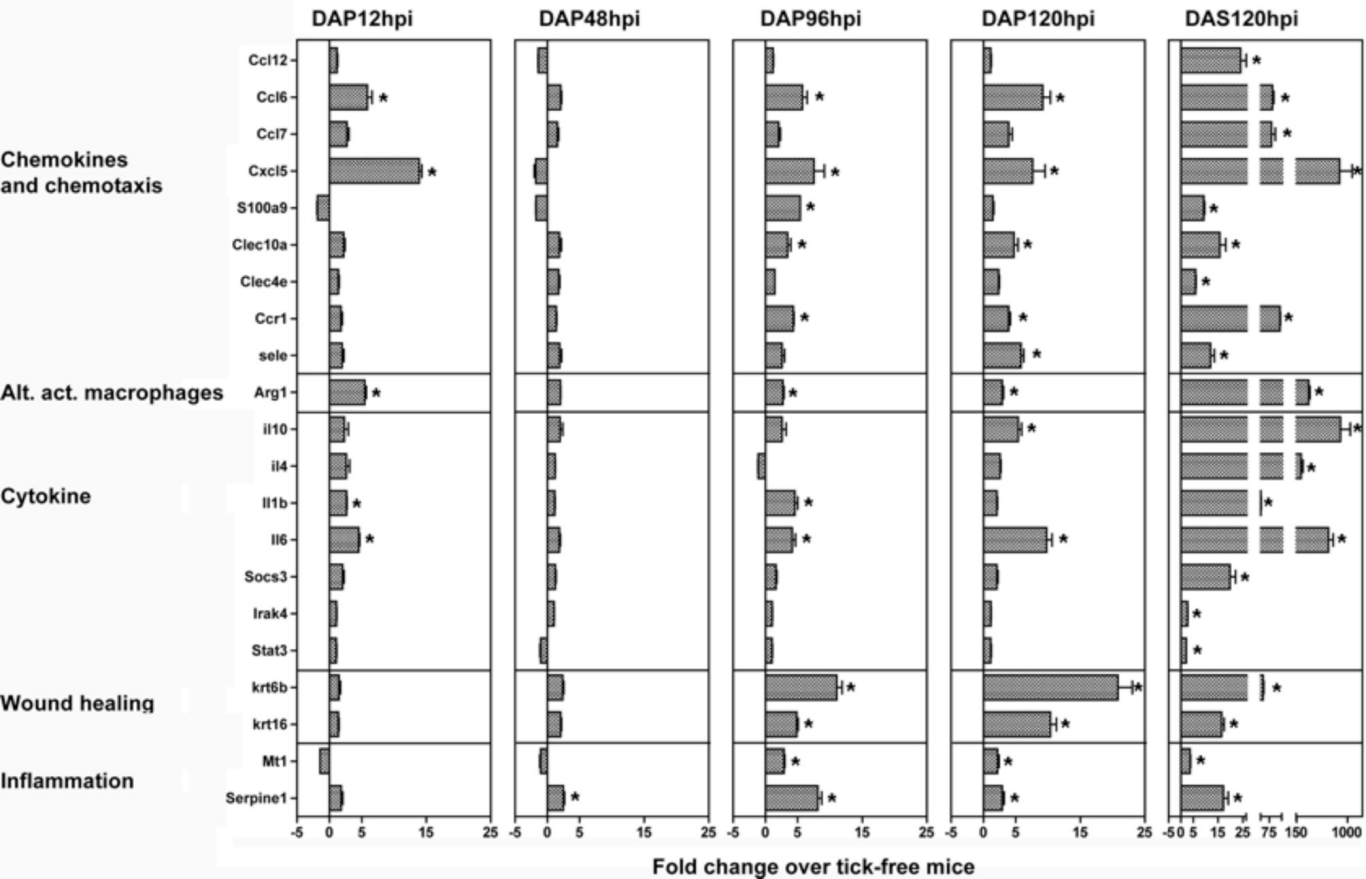
**Validation of microarray data by quantitative real-time PCR**. A list of genes significantly modulated in the microarray study (Table 2) were validated using quantitative real-time PCR on bite site skin samples from a separate infestation experiment. Significance was assessed using LIMMA (linear models in microarray analysis) implemented in HTqPCR, and R based program for qrt-PCR analysis (see methods chapter). All the genes marked with an asterisk were significant (*p* < 0.05) as compared to tick-free mice.

A list of genes was chosen to validate the microarray data (Table 2) using quantitative real-time PCR. Genes were chosen based on significant fold change in the microarray study that were involved in important pathways identified in the gene ontology analysis. Trends of up- and downregulation were well preserved between the microarray and validation study (Figure 4). In general, more genes had significant fold changes in the microarray that the validation for 12 and 48 hpi, and the validation had more significant fold changes from 96 hpi onwards. At the 120 hpi secondary infestation time point, all the gene targets validated except Chi3l1 and Wnk1. It should be noted that some genes significant only at later time points in the array study became significant earlier in the validation study (i.e., Arg1, Cxcl5), while some genes significant early in the array study did not become significant until later time points in the validation (i.e., Ccl12, Ccl7, Krt6b, Krt16, and S100a9). Interestingly, the trend of more significant gene modulation at 12 than 48 hpi seen in the array data was upheld in the validation study.

In summary, the validation study upheld the microarray study with a few minor differences. Samples used for the validation study were derived from a completely separate infestation experiment than the array study, making it an extremely rigorous test and likely contributing to the small variations seen between data sets.

### Lymph node study

**Figure 5:**
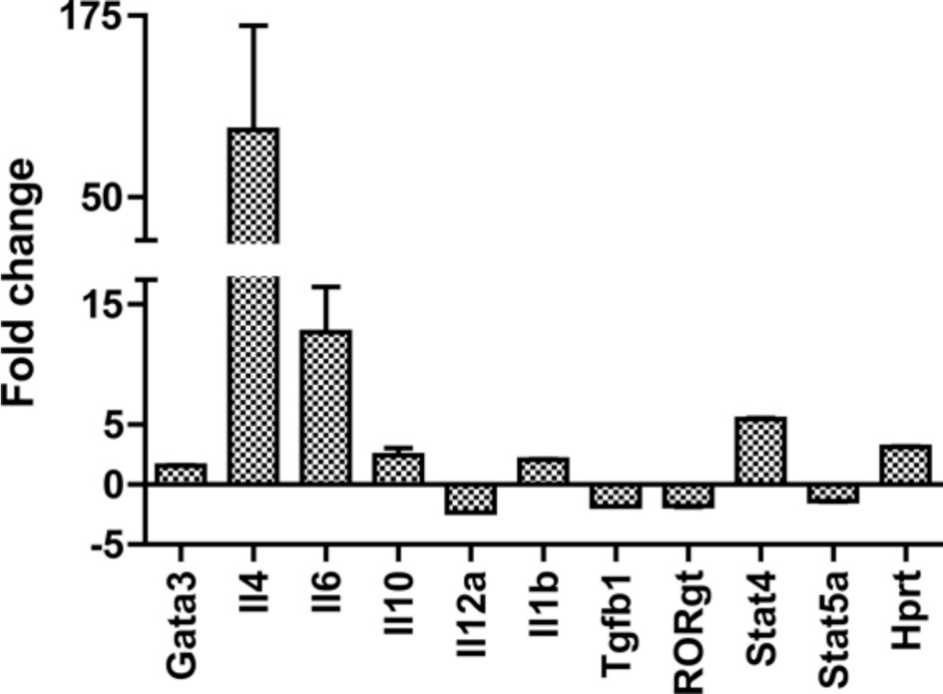
**Lymph node gene expression profile supports a systemic Th2 response**. Gene expression changes between secondary infestation 120 hpi draining lymph nodes and lymph nodes from tick-free mice measured by quantitative real-time PCR. Significance was assessed using LIMMA (linear models in microarray analysis) implemented in HTqPCR, and R based program for qrt-PCR analysis (see methods chapter). All the genes shown were significant (*p* < 0.05).

The host response to ticks has frequently been reported to be polarized toward the production of Th2 cytokines. However, in our earlier work with *I. scapularis*, the gene expression profile in the skin was consistent with a more mixed response (Heinze et al., 2012a,b). Consistent with these results, gene expression profiling in the skin at *D. andersoni* bites did not yield a conclusive picture of CD4+ T cell polarization. While IL-4 was present, so were IL-6 and IL-1b, cytokines more associated with Th17 responses than Th2. To assay the systemic host response, the expression of a panel of genes (Table 4) related to CD4+ T cell differentiation was measured in lymph node samples from naïve and 120 hpi secondary infestation mice. Important transcription factors for T regulatory cells (Foxp3), Th17 cells (Rorgt), Th1 cells (Tbx21) and Th1 cytokine (IL-12a) were either downregulated or unchanged between infested and control mice. In contrast, the Th2 cytokine IL-4 was upregulated (Figure 5). Thus, the results of this experiment strongly suggest the systemic response to repeated *D. andersoni* nymphal feeding in mice is polarized toward a Th2 profile.

### Histology

**Figure 6:**
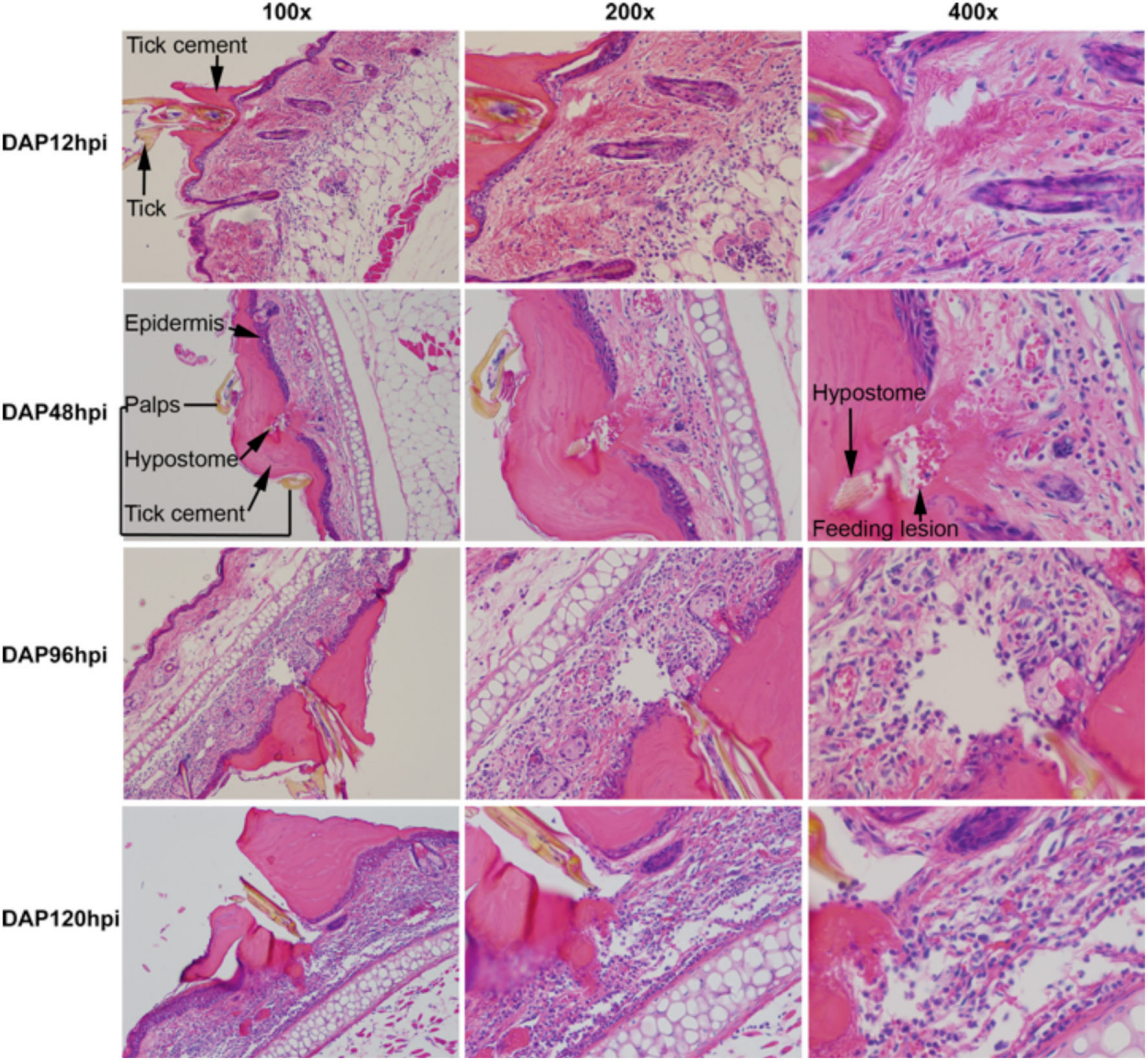
**Histological analysis of *D. andersoni* bites during primary infestations**. Biopsies from tick feeding sites were fixed in zinc fixative and embedded in paraffin. Paraffin blocks were carefully sectioned and slides showing the entry of the hypostome into the skin were stained with H&E. In the top two rows, relevant structures such as the tick, tick cement, epidermis, palps, and feeding lesion have been labeled. The second row appears different that the others because the orientation of the tick in the block was different.

The bite site is characterized by a large quantity of attachment cement secreted by the tick to anchor it to the host (Figure 6). The amount of cement increases throughout the feeding period, and it contains readily evident layers, suggesting that additional cement is secreted in waves throughout the feeding cycle. The deepest penetration of the hypostome into the skin is very shallow, in most cases barely passing through the epidermis. At 12 hpi, a mild inflammatory infiltrate was present in the dermis, especially concentrated at the junction between dermal connective tissue and underlying adipose tissue. A small lesion was located immediately under the hypostome and likely represents the feeding “pool.” At 48 hpi, the number of inflammatory cells had not increased from 12 hpi, mirroring the reduction in gene expression seen at this time point. The feeding lesion is very well defined, and extravasated erythrocytes are readily evident around the hypostome. Some thickening of the epidermis is evident. By 96 hpi, the inflammatory infiltrate has increased dramatically and the feeding lesion appears to have moved deeper into the dermis. Epidermal thickening and extravasated erythrocytes and leukocytes are present. At 120 hpi, most of the changes at 96 hpi are intensified. The infiltrate is very dense, the epidermis is markedly thickened, the feeding lesion is poorly defined and the dermal tissue near the hypostome appears to be loosing its normal architecture. Extravasated erythrocytes and leukocytes are prominent. In the microarray, the chemotaxis of immune cells into the bite site, and aspects of wound healing responses were especially prominent. The histological analysis supports these basic conclusions, showing an inflammatory infiltrate that increases across time, and significant changes in the epidermal and dermal compartments near the feeding tick.

## Conclusions

The microarray data suggests the chemotaxis of neutrophils, monocytes, and other cell types, the production and scavenging of reactive oxygen species, keratin-based wound healing responses, and C-type lectins are important host responses to tick feeding conserved across primary and secondary infestations. Early time points in the primary infestation imply the inhibition of transcription and RNA processing. This inhibitory profile may represent tick-induced modulation of early host responses. Later time points in the primary infestation provide additional support for wound healing activities including the suggestion of EMT. The secondary infestation is characterized by increases in these same pathways, along with the addition of complement, coagulation, Toll-like receptors, acute phase response, antigen presentation, and the activation of lymphocytes. These results were supported by quantitative real-time PCR validation. The systemic murine response to *D. andersoni* nymphal feeding was polarized toward a Th2, based on real-time PCR analysis of draining lymph nodes from secondary infestations. The histological analysis supported the chemotaxis of neutrophils and monocytes into the bite site and the marked thickening of the epithelial layers may be the result of the upregulation of genes related to keratinization and wound healing. In conclusion, during *D. andersoni* feeding infiltration of inflammatory cells increases across time concurrent with significant changes in the epidermal and dermal compartments near the feeding tick. The importance of changes in the epidermal layer in the host response to ticks is not known, however, it is possible the host attempts to “slough off” the tick by greatly increasing epithelial cell replication.

### Conflict of interest statement

The authors declare that the research was conducted in the absence of any commercial or financial relationships that could be construed as a potential conflict of interest.

## References

[B1] Abreu-Blanco M. T. Watts J. J. Verboon J. M. Parkhurst S. M. (2012). Cytoskeleton responses in wound repair. Cell. Mol. Life Sci. 69, 2469–2483 10.1007/s00018-012-0928-2 22349211 PMC3388155

[B2] Akira S. Takeda K. (2004). Toll-like receptor signalling. Nat. Rev. Immunol. 4, 499–511 10.1038/nri1391 15229469

[B3] Andoh T. Yoshida T. Lee J. B. Kuraishi Y. (2012). Cathepsin E induces itch-related response through the production of endothelin-1 in mice. Eur. J. Pharmacol. 686, 16–21 10.1016/j.ejphar.2012.04.024 22546226

[B4] Barrett N. A. Maekawa A. Rahman O. M. Austen K. F. Kanaoka Y. (2009). Dectin-2 recognition of house dust mite triggers cysteinyl leukotriene generation by dendritic cells. J. Immunol. 182, 1119–1128 10.4049/jimmunol.182.2.1119 19124755 PMC3682801

[B5] Beaulieu E. Morand E. F. (2011). Role of GILZ in immune regulation, glucocorticoid actions and rheumatoid arthritis. Nat. Rev. Rheumatol. 7, 340–348 10.1038/nrrheum.2011.59 21556028

[B6] Bouchard K. R. Wikel S. K. (2005). Care, maintenance, and experimental infestation of ticks in the laboratory setting, in Biology of Disease Vectors, 2nd Edn., ed Marquart W. C. (San Diego, CA: Elsevier Academic Press), 705–711

[B7] Diczfalusy U. Olofsson K. E. Carlsson A. M. Gong M. Golenbock D. T. Rooyackers O. (2009). Marked upregulation of cholesterol 25-hydroxylase expression by lipopolysaccharide. J. Lipid Res. 50, 2258–2264 10.1194/jlr.M900107-JLR200 19502589 PMC2759831

[B8] Durr H. Flaus A. Owen-Hughes T. Hopfner K. P. (2006). Snf2 family ATPases and DExx box helicases: differences and unifying concepts from high-resolution crystal structures. Nucleic Acids Res. 34, 4160–4167 10.1093/nar/gkl540 16935875 PMC1616948

[B9] Dvinge H. Bertone P. (2009). HTqPCR: high-throughput analysis and visualization of quantitative real-time PCR data in R. Bioinformatics 25 3325–3326 10.1093/bioinformatics/btp578 19808880 PMC2788924

[B10] Geijtenbeek T. B. Gringhuis S. I. (2009). Signalling through C-type lectin receptors: shaping immune responses. Nat. Rev. Immunol. 9, 465–479 10.1038/nri2569 19521399 PMC7097056

[B11] Gery S. Komatsu N. Baldjyan L. Yu A. Koo D. Koeffler H. P. (2006). The circadian gene per1 plays an important role in cell growth and DNA damage control in human cancer cells. Mol. Cell 22, 375–382 10.1016/j.molcel.2006.03.038 16678109

[B12] Heinze D. M. Carmical J. R. Aronson J. F. Thangamani S. (2012a). Early immunologic events at the tick-host interface. PLoS ONE 7:e47301 10.1371/journal.pone.0047301 23077588 PMC3471850

[B13] Heinze D. M. Wikel S. K. Thangamani S. Alarcon-Chaidez F. J. (2012b). Transcriptional profiling of the murine cutaneous response during initial and subsequent infestations with *Ixodes scapularis* nymphs. Parasit. Vectors 5:26 10.1186/1756-3305-5-26 22309607 PMC3293053

[B14] Huang da W. Sherman B. T. Lempicki R. A. (2009a). Bioinformatics enrichment tools: paths toward the comprehensive functional analysis of large gene lists. Nucleic Acids Res. 37, 1–13 10.1093/nar/gkn923 19033363 PMC2615629

[B15] Huang da W. Sherman B. T. Lempicki R. A. (2009b). Systematic and integrative analysis of large gene lists using DAVID bioinformatics resources. Nat. Protoc. 4, 44–57 10.1038/nprot.2008.211 19131956

[B16] Hovius J. W. de Jong M. A. den Dunnen J. Litjens M. Fikrig E. van der Poll T. (2008). Salp15 binding to DC-SIGN inhibits cytokine expression by impairing both nucleosome remodeling and mRNA stabilization. PLoS Pathog. 4:e31 10.1371/journal.ppat.0040031 18282094 PMC2242833

[B17] Inoue K. Takano H. Shimada A. Satoh M. (2009). Metallothionein as an anti-inflammatory mediator. Mediators Inflamm. 2009:101659 10.1155/2009/101659 19436762 PMC2679981

[B18] Kahle K. T. Ring A. M. Lifton R. P. (2008). Molecular physiology of the WNK kinases. Annu. Rev. Physiol. 70, 329–355 10.1146/annurev.physiol.70.113006.100651 17961084

[B19] Kazimírová M. Štibrániová I. (2013). Tick salivary compounds: their role in modulation of host defences and pathogen transmission. Front. Cell. Infect. Microbiol. 20:43 10.3389/fcimb.2013.00043 23971008 PMC3747359

[B20] Kuester D. Lippert H. Roessner A. Krueger S. (2008). The cathepsin family and their role in colorectal cancer. Pathol. Res. Pract. 204, 491–500 10.1016/j.prp.2008.04.010 18573619

[B21] Leopold P. L. Vincent J. Wang H. (2012). A comparison of epithelial-to-mesenchymal transition and re-epithelialization. Semin. Cancer Biol. 22, 471–483 10.1016/j.semcancer.2012.07.003 22863788 PMC3595494

[B22] Lund E. G. Kerr T. A. Sakai J. Li W. P. Russell D. W. (1998). cDNA cloning of mouse and human cholesterol 25-hydroxylases, polytopic membrane proteins that synthesize a potent oxysterol regulator of lipid metabolism. J. Biol. Chem. 273, 34316–34327 10.1074/jbc.273.51.34316 9852097

[B23] Mans B. J. de Klerk D. Pienaar R. de Castro M. H. Latif A. A. (2012). The mitochondrial genomes of nuttalliella namaqua (Ixodoidea: Nuttalliellidae) and argas africolumbae (Ixodoidae: Argasidae): estimation of divergence dates for the major tick lineages and reconstruction of ancestral blood-feeding characters. PLoS ONE 7:e49461 10.1371/journal.pone.0049461 23145176 PMC3493528

[B24] Moser T. S. Schieffer D. Cherry S. (2012). AMP-activated kinase restricts rift valley fever virus infection by inhibiting fatty acid synthesis. PLoS Pathog. 8:e1002661 10.1371/journal.ppat.1002661 22532801 PMC3330235

[B25] Nakamura M. Tokura Y. (2011). Epithelial-mesenchymal transition in the skin. J. Dermatol. Sci. 61, 7–13 10.1016/j.jdermsci.2010.11.015 21167690

[B26] Oliveira C. J. Carvalho W. A. Garcia G. R. Gutierrez F. R. de Miranda Santos I. K. Silva J. S. (2010). Tick saliva induces regulatory dendritic cells: MAP-kinases and Toll-like receptor-2 expression as potential targets. Vet. Parasitol. 167, 288–297 10.1016/j.vetpar.2009.09.031 19836139

[B27] Oliveros J. C. (2007). VENNY. An Interactive Tool for Comparing Lists With Venn Diagrams. Available online at: http://bioinfogp.cnb.csic.es/tools/venny/index.html (Accessed Aug, 2012).

[B28] Paladini R. D. Takahashi K. Bravo N. S. Coulombe P. A. (1996). Onset of re-epithelialization after skin injury correlates with a reorganization of keratin filaments in wound edge keratinocytes: defining a potential role for keratin 16. J. Cell Biol. 132, 381–397 10.1083/jcb.132.3.381 8636216 PMC2120730

[B29] Patel S. Kartasova T. Segre J. A. (2003). Mouse Sprr locus: a tandem array of coordinately regulated genes. Mamm. Genome 14, 140–148 10.1007/s00335-002-2205-4 12584609

[B30] Sakuma S. Kitamura T. Kuroda C. Takeda K. Nakano S. Hamashima T. (2012). All-trans arachidonic acid generates reactive oxygen species via xanthine dehydrogenase/xanthine oxidase interconversion in the rat liver cytosol *in vitro*. J. Clin. Biochem. Nutr. 51, 55–60 10.3164/jcbn.11-97 22798714 PMC3391864

[B31] Schroeder A. Mueller O. Stocker S. Salowsky R. Leiber M. Gassmann M. (2006). The RIN: an RNA integrity number for assigning integrity values to RNA measurements. BMC Mol. Biol. 7:3 10.1186/1471-2199-7-3 16448564 PMC1413964

[B32] Thirumoorthy N. Shyam Sunder A. Manisenthil Kumar K. Senthil Kumar M. Ganesh G. Chatterjee M. (2011). A review of metallothionein isoforms and their role in pathophysiology. World J. Surg. Oncol. 9:54 10.1186/1477-7819-9-54 21599891 PMC3114003

[B33] Titus R. Bishop J. Mejia J. (2006). The immunomodulatory factors of arthropod saliva and the potential for these factors to serve as vaccine targets to prevent pathogen transmission. Parasite Immunol. 28, 131–141 10.1111/j.1365-3024.2006.00807.x 16542315

[B34] Ugajin T. Satoh T. Kanamori T. Aritake K. Urade Y. Yokozeki H. (2011). FcepsilonRI, but not FcgammaR, signals induce prostaglandin D2 and E2 production from basophils. Am. J. Pathol. 179, 775–782 10.1016/j.ajpath.2011.04.023 21712025 PMC3157207

[B35] Warnes G. R. Bolker B. Bonebakker L. Gentleman R. Huber W. Liaw A. (2013). GPLOTS: Various R Programming Tools for Plotting Data. Aviiable online at: http://CRAN.R-project.org/package=gplots R package version 2.12.11.

[B36] Wikel S. (2013). Ticks and tick-borne pathogens at the cutaneous interface: host defenses, tick countermeasures, and a suitable environment for pathogen establishment. Front. Microbiol. 4:337 10.3389/fmicb.2013.00337 24312085 PMC3833115

[B37] Wong P. Coulombe P. A. (2003). Loss of keratin 6 (K6) proteins reveals a function for intermediate filaments during wound repair. J. Cell Biol. 163, 327–337 10.1083/jcb.200305032 14568992 PMC2173512

[B38] Yamamoto K. Kawakubo T. Yasukochi A. Tsukuba T. (2012). Emerging roles of cathepsin E in host defense mechanisms. Biochim. Biophys. Acta 1824, 105–112 10.1016/j.bbapap.2011.05.022 21664991

[B39] Zou T. Garifulin O. Berland R. Boyartchuk V. L. (2011). *Listeria monocytogenes* infection induces prosurvival metabolic signaling in macrophages. Infect. Immun. 79, 1526–1535 10.1128/IAI.01195-10 21263022 PMC3067555

